# Trends in Medical Imaging During Pregnancy in the United States and Ontario, Canada, 1996 to 2016

**DOI:** 10.1001/jamanetworkopen.2019.7249

**Published:** 2019-07-24

**Authors:** Marilyn L. Kwan, Diana L. Miglioretti, Emily C. Marlow, E. J. Aiello Bowles, Sheila Weinmann, Stephanie Y. Cheng, Kamala A. Deosaransingh, Prachi Chavan, Lisa M. Moy, Wesley E. Bolch, James R. Duncan, Robert T. Greenlee, Lawrence H. Kushi, Jason D. Pole, Alanna K. Rahm, Natasha K. Stout, R. Smith-Bindman

**Affiliations:** 1 Division of Research, Kaiser Permanente Northern California, Oakland; 2 Department of Public Health Sciences, University of California, Davis; 3 Kaiser Permanente Washington Health Research Institute, Kaiser Permanente Washington, Seattle; 4 Center for Health Research, Kaiser Permanente Northwest, Portland, Oregon; 5 Center for Health Research, Kaiser Permanente Hawaii, Honolulu; 6 ICES, Toronto, Ontario, Canada; 7 Department of Radiology and Biomedical Imaging, University of California, San Francisco; 8 Department of Biomedical Engineering, University of Florida, Gainesville; 9 Interventional Radiology Section, Washington University in St Louis, St Louis, Missouri; 10 Marshfield Clinic Research Institute, Marshfield Clinic Health System, Marshfield, Wisconsin; 11 Pediatric Oncology Group of Ontario, Toronto, Ontario, Canada; 12 Dalla Lana School of Public Health, University of Toronto, Toronto, Ontario, Canada; 13 Center for Health Research, Genomic Medicine Institute, Geisinger, Danville, Pennsylvania; 14 Department of Population Medicine, Harvard Medical School, Harvard Pilgrim Health Care Institute, Boston, Massachusetts; 15 Department of Epidemiology and Biostatistics, University of California, San Francisco; 16 Department of Obstetrics, Gynecology, and Reproductive Sciences, University of California, San Francisco

## Abstract

**Importance:**

The use of medical imaging has sharply increased over the last 2 decades. Imaging rates during pregnancy have not been quantified in a large, multisite study setting.

**Objective:**

To evaluate patterns of medical imaging during pregnancy.

**Design, Setting, and Participants:**

A retrospective cohort study was performed at 6 US integrated health care systems and in Ontario, Canada. Participants included pregnant women who gave birth to a live neonate of at least 24 weeks’ gestation between January 1, 1996, and December 31, 2016, and who were enrolled in the health care system for the entire pregnancy.

**Exposures:**

Computed tomography (CT), magnetic resonance imaging, conventional radiography, angiography and fluoroscopy, and nuclear medicine.

**Main Outcomes and Measures:**

Imaging rates per pregnancy stratified by country and year of child’s birth.

**Results:**

A total of 3 497 603 pregnancies in 2 211 789 women were included. Overall, 26% of pregnancies were from US sites. Most (92%) were in women aged 20 to 39 years, and 85% resulted in full-term births. Computed tomography imaging rates in the United States increased from 2.0 examinations/1000 pregnancies in 1996 to 11.4/1000 pregnancies in 2007, remained stable through 2010, and decreased to 9.3/1000 pregnancies by 2016, for an overall increase of 3.7-fold. Computed tomography rates in Ontario, Canada, increased more gradually by 2.0-fold, from 2.0/1000 pregnancies in 1996 to 6.2/1000 pregnancies in 2016, which was 33% lower than in the United States. Overall, 5.3% of pregnant women in US sites and 3.6% in Ontario underwent imaging with ionizing radiation, and 0.8% of women at US sites and 0.4% in Ontario underwent CT. Magnetic resonance imaging rates increased steadily from 1.0/1000 pregnancies in 1996 to 11.9/1000 pregnancies in 2016 in the United States and from 0.5/1000 pregnancies in 1996 to 9.8/1000 pregnancies in 2016 in Ontario, surpassing CT rates in 2013 in the United States and in 2007 in Ontario. In the United States, radiography rates doubled from 34.5/1000 pregnancies in 1996 to 72.6/1000 pregnancies in 1999 and then decreased to 47.6/1000 pregnancies in 2016; rates in Ontario slowly increased from 36.2/1000 pregnancies in 1996 to 44.7/1000 pregnancies in 2016. Angiography and fluoroscopy and nuclear medicine use rates were low (5.2/1000 pregnancies), but in most years, higher in Ontario than the United States. Imaging rates were highest for women who were younger than 20 years or aged 40 years or older, gave birth preterm, or were black, Native American, or Hispanic (US data only). Considering advanced imaging only, chest imaging of pregnant women was more likely to use CT in the United States and nuclear medicine imaging in Ontario.

**Conclusions and Relevance:**

The use of CT during pregnancy substantially increased in the United States and Ontario over the past 2 decades. Imaging rates during pregnancy should be monitored to avoid unnecessary exposure of women and fetuses to ionizing radiation.

## Key Points

**Question:**

What are the trends over time in the use of medical imaging (computed tomography, magnetic resonance imaging, angiography and fluoroscopy, and nuclear medicine) during pregnancy in the United States and Ontario, Canada?

**Findings:**

In this cohort study conducted over a 21-year follow-up period (1996-2016), computed tomography use rates increased by 3.7-fold in US sites and 2.0-fold in Ontario, whereas use rates for other imaging modalities with ionizing radiation decreased. Overall, 5.3% of pregnant women in US sites and 3.6% in Ontario underwent imaging with ionizing radiation, and 0.8% in US sites and 0.4% in Ontario underwent computed tomography.

**Meaning:**

The use of computed tomography during pregnancy has increased substantially over the past 2 decades and should be monitored to avoid unnecessary exposure of women and fetuses to ionizing radiation.

## Teaser

This cohort study evaluates trends of medical imaging, including computed tomography, magnetic resonance imaging, angiography and fluoroscopy, and nuclear medicine, among pregnant women in the United States and Ontario, Canada.

## Introduction

It is widely acknowledged that ionizing radiation exposure is associated with potential carcinogenic, teratogenic, and mutagenic health risks to the developing fetus.^1,2,3,4^ These health risks can translate into congenital abnormalities, developmental delays, and cancer. The characterization and severity of these fetal risks depend on the gestational age at the time of exposure and radiation dose to the fetus.^5^ Because of the potential repercussions, ultrasonography replaced abdominal radiographs several decades ago for monitoring pregnancy-related complications. However, as reported in a single-institution US study,^6^ the use of computed tomography (CT) and nuclear medicine imaging might still be increasing in pregnant women, with these modalities delivering a far higher exposure to ionizing radiation than conventional radiography.

Several reviews^1,7,8^ have quantified the potential harm of radiation exposure in pregnant women and concluded that exposure to radiation from medical imaging should be carefully considered while weighing risks and benefits to the patient. However, professional society recommendations^5,9^ over the last 2 decades have not consistently recommended minimizing medical imaging during pregnancy. Notably, a CT scan of the maternal abdomen and pelvis delivers a dose of 15 to 21 mGy to the fetus,^10^ similar to doses associated with increased leukemia risk in children.^11^ The guidelines emphasize that medical radiation likely delivers doses below deterministic thresholds for causing fetal effects. However, some risks, such as cancer induction, are thought to be proportional to fetal doses with no threshold for potential harm.^1,12^

We conducted a population-based assessment of medical imaging use over the period 1996 to 2016 for pregnant women who gave birth to a live neonate enrolled in 6 US integrated health care systems or who were residing in Ontario, Canada, a province with a single-payer health system. We compared trends in the use of major imaging modalities other than ultrasonography, including CT, angiography and fluoroscopy, magnetic resonance imaging (MRI), conventional radiography, and nuclear medicine. Understanding imaging use for pregnant women may help characterize the variability and potential overuse of imaging with ionizing radiation in this patient population and inform the quantification of ionizing radiation exposure that poses fetal risks.

## Methods

Data from 7 Radiation-Induced Cancers Study collaborators were included in this analysis.^13^ The 6 US sites were the Kaiser Permanente regions of Northern California, Northwest, Washington (formerly Group Health Cooperative until February 2017), and Hawaii; Marshfield Clinic Health System in Wisconsin; and Harvard Pilgrim Health Care in Massachusetts. Each Kaiser Permanente region operates independently. To be eligible, pregnant women had to give birth to a live neonate of at least 24 weeks’ gestational age between January 1, 1996, and December 31, 2016 (January 1, 2000, to December 31, 2016, for Harvard Pilgrim). To ensure complete capture of medical imaging, mothers were required to be enrolled in the health system for their entire pregnancy. We adhered to the Strengthening the Reporting of Observational Studies in Epidemiology (STROBE) reporting guideline in the reporting of our results and discussion.^14^ This study was approved by institutional review boards at each participating study site, including University of California, San Francisco and University of California, Davis. Participant informed consent was waived because the data were deidentified.

### Data Sources

For US sites, complete medical diagnoses and imaging use data were available through the Virtual Data Warehouse, a collaborative data model structure developed by the National Cancer Institute–supported Cancer Research Network and Health Care Systems Research Network.^15,16^ Data were obtained from each site’s clinical and administrative data sources, including the electronic health record, through distributive programs developed at 1 site. For Ontario, complete medical diagnoses and imaging use data were ascertained through physician billing records available in the Ontario Health Insurance Plan database, hospital discharge records available in the Discharge Abstract Database, and emergency department visits available in the National Ambulatory Care Reporting System. The Canadian data sets were linked using unique encoded identifiers.^17^ Established quality assurance methods, including targeted medical record review within and across the study sites, were used to ensure complete capture of imaging use.^18^

To identify eligible pregnancies with live births in the United States, data were obtained from birth registries, linkages to state birth certificate data, and/or *International Classification of Diseases, Ninth Revision* and *Tenth Revision* and *Current Procedural Terminology* live birth codes. Gestational age from last menstrual cycle to birth was collected from US site-specific birth registries (4 sites) or birth certificate data (1 site) and was unavailable for 1 site. In Ontario, newborn birth inpatient Discharge Abstract Database records were linked to maternal inpatient Discharge Abstract Database records to obtain gestational age.^19^ Gestational age was assumed to be 40 weeks if missing. The start of pregnancy was estimated on the basis of the child’s birthdate and gestational age at birth. If gestational age at birth was less than 24 weeks, the pregnancy was excluded because this was considered a likely nonviable pregnancy. Pregnancies with unknown maternal age at the time of birth were excluded (593 women). Maternal race and ethnicity from self-report were available only for US sites.

### Imaging Use

Dates and types of all imaging examinations during pregnancy were collected. At the US sites, imaging procedures were captured primarily using standardized *International Classification of Diseases, Ninth Revision* and *Tenth Revision*, *Current Procedural Terminology*, and Healthcare Common Procedure Coding System codes^20^; some site-specific legacy codes were mapped to these codes. Code modifiers for the technical, physician, or global components and examinations irrespective of the physician specialty were included. Some billing codes changed over time, and all codes were mapped to an anatomic area and modality to ensure consistency over time, updating previously used codes.^21^ For Ontario, Canadian Classification of Health Intervention codes in the Discharge Abstract Database and National Ambulatory Care Reporting System and fee codes in the Ontario Health Insurance Plan were mapped to the equivalent or closest US code.

Across all years, we captured 4774 unique imaging codes, each mapped to an imaging modality (CT, MRI, radiography, angiography, fluoroscopy, nuclear medicine, ultrasonography, and other, including unknown) and anatomic area (abdomen or pelvis, chest, head or brain, extremity, neck, spine, unknown, and other for nuclear medicine). Imaging procedures performed in conjunction with radiation treatment for cancer, biopsies, image processing, or reinterpretation of images were excluded. Multiple examinations of the same modality and anatomic area performed of the same patient on the same day were counted only once to reduce the likelihood of overcounting duplicate codes. Given that we used the conventional pregnancy dating of time since last menstrual period, examinations within the first 14 days of pregnancy were excluded as likely occurring before conception. We excluded examinations that occurred after the delivery date and time.

Analyses focused on CT, MRI, radiography, angiography and fluoroscopy, and nuclear medicine. Angiography and fluoroscopy were combined because both use continuous x-rays and have relatively similar radiation exposures, and angiography reflects a relatively small percentage (11%) of examination types. Ultrasonography was excluded because of inconsistencies across sites in the capture of examinations conducted during routine prenatal care, because these may be performed by obstetrician-gynecologists. Nuclear medicine was subdivided into ventilation-perfusion (V/Q) scanning and all other nuclear medicine imaging (planar imaging, single-photon emission CT, and positron emission tomography).

### Statistical Analysis

Analyses were at the level of the pregnancy. We summarized the distribution and percentage of pregnancies with any imaging for each modality by demographic factors. For each modality and anatomic area, we calculated the imaging rate per pregnancy by country and year. Rates in the United States were averaged across health systems, weighting each health system equally given the differences in sample sizes across the US sites. Specifically, 1 health care system was very large, and we did not want the rates to be heavily weighted toward that system. Multivariable Poisson regression was used to estimate adjusted relative rates (RRs) of imaging and 95% confidence intervals, stratified by modality, associated with a priori system-level and person-level variables of interest that might be associated with imaging, while also considering data availability across study sites. Variables included study site, maternal age at the start of pregnancy, gestational age of the neonate at birth, and year of the neonate’s birth. The variance of the Poisson distribution was inflated by a scale parameter, estimated as the deviance divided by the degrees of freedom, to allow for overdispersion. A separate model was fit to US sites to estimate RRs associated with maternal race and ethnicity, adjusting for the other covariates. The 95% confidence intervals not including 1.0 were considered to indicate statistically significant differences in RRs.

To contrast the choice of imaging modalities used at US sites and Ontario, we calculated imaging rates for chest imaging, surmising that most of these examinations in women of childbearing age reflected imaging for suspected pulmonary embolism.^22,23^ We compared the 2 most common modalities for chest imaging, chest CT and nuclear medicine V/Q scanning, and evaluated differences over time and trimesters defined as first (1-12 weeks), second (13-26 weeks), and third (>27 weeks).

## Results

A total of 3 497 603 pregnancies in 2 211 789 women were included (Table 1). Overall, 26% of pregnancies were from US sites. Most (92%) were in women aged 20 to 39 years, and 85% resulted in full-term births.

**Table 1.** Characteristics of 3 497 603 Pregnancies Among 2 211 789 Women, Overall and by Imaging Modality, US Sites and Ontario, Canada^**a**^

Variable	Unique Pregnancies	Pregnancies by Imaging Modality^b^				
		Computed Tomography	Magnetic Resonance Imaging	Radiography	Fluoroscopy and Angiography	Nuclear Medicine
Pregnancies at US site	3 497 603	16 267 (0.47)	14 625 (0.42)	122 174 (3.49)	11 007 (0.31)	6904 (0.20)
1	23 217 (0.66)	259 (1.12)	139 (0.60)	1853 (7.98)	115 (0.50)	80 (0.34)
2	37 975 (1.09)	220 (0.58)	159 (0.42)	3042 (8.01)	72 (0.19)	63 (0.17)
3	77 541 (2.22)	599 (0.77)	487 (0.63)	3316 (4.28)	204 (0.26)	151 (0.19)
4	79 774 (2.28)	670 (0.84)	594 (0.74)	4090 (5.13)	314 (0.39)	141 (0.18)
5	101 513 (2.90)	989 (0.97)	1463 (1.44)	4183 (4.12)	459 (0.45)	276 (0.27)
6	587 814 (16.81)	4038 (0.69)	2334 (0.40)	25 497 (4.34)	1024 (0.17)	653 (0.11)
Pregnancies in Ontario, Canada	2 589 769 (74.04)	9492 (0.37)	9449 (0.36)	80 193 (3.10)	8819 (0.34)	5540 (0.21)
Maternal age at start of pregnancy, y	3 497 603	16 267	14 625	122 174	11 007	6904
≤19	188 451 (5.39)	1239 (0.66)	648 (0.34)	9974 (5.29)	629 (0.33)	456 (0.24)
20-29	1 572 292 (44.95)	7792 (0.50)	5974 (0.38)	55 230 (3.51)	4841 (0.31)	3258 (0.21)
30-39	1 647 359 (47.10)	6720 (0.41)	7399 (0.45)	52 261 (3.17)	5120 (0.31)	2966 (0.18)
≥40	89 501 (2.56)	516 (0.58)	604 (0.67)	4709 (5.26)	417 (0.47)	224 (0.25)
Gestational age of neonate at birth, wk^c^	3 497 603	16 267	14 625	122 174	11 007	6904
24-36 (Preterm)	188 272 (5.38)	1863 (0.99)	2282 (1.21)	10 310 (5.48)	1287 (0.68)	595 (0.32)
37-40 (Full-term)	2 973 888 (85.03)	13 150 (0.44)	11 329 (0.38)	101 044 (3.40)	9017 (0.30)	5787 (0.19)
41-43 (Late-term)	335 443 (9.59)	1254 (0.37)	1014 (0.30)	10 820 (3.23)	703 (0.21)	522 (0.16)
Year of neonate’s birth	3 497 603	16 267	14 625	122 174	11 007	6904
1996-2000	756 497 (21.63)	1888 (0.25)	808 (0.11)	25 765 (3.41)	2950 (0.39)	1126 (0.15)
2001-2005	826 469 (23.63)	3363 (0.41)	2040 (0.25)	28 992 (3.51)	2427 (0.29)	1638 (0.20)
2006-2010	873 254 (24.97)	4829 (0.55)	4035 (0.46)	29 974 (3.43)	2393 (0.27)	1847 (0.21)
2011-2016	1 041 383 (29.77)	6187 (0.59)	7742 (0.74)	37 443 (3.60)	3237 (0.31)	2293 (0.22)
Race of mother at US sites^d^	907 834	6775	5176	41 981	2188	1364
White	443 222 (48.82)	3270 (0.74)	2628 (0.59)	19 679 (4.44)	1171 (0.26)	626 (0.14)
Asian	166 867 (18.38)	766 (0.46)	521 (0.31)	7955 (4.77)	243 (0.15)	174 (0.10)
Black	55 056 (6.06)	823 (1.49)	356 (0.65)	3683 (6.69)	150 (0.27)	126 (0.23)
Hawaiian/Pacific Islander	14 620 (1.61)	114 (0.78)	65 (0.44)	847 (5.79)	34 (0.23)	22 (0.15)
Native American	5581 (0.61)	65 (1.16)	41 (0.73)	329 (5.90)	16 (0.29)	9 (0.16)
Unknown	222 488 (24.51)	1737 (0.78)	1565 (0.70)	9488 (4.26)	574 (0.26)	407 (0.18)
Ethnicity of mother at US sites^d^	907 834	6775	4132	41 981	2188	1364
Not Hispanic	576 984 (63.56)	4101 (0.71)	2993 (0.52)	26 699 (4.63)	1289 (0.22)	807 (0.14)
Hispanic	172 658 (19.02)	1384 (0.80)	777 (0.45)	8134 (4.71)	319 (0.18)	195 (0.11)
Unknown or not reported	158 192 (17.43)	1290 (0.82)	362 (0.23)	7148 (4.52)	580 (0.37)	362 (0.23)

^a^ Data are number (%) of pregnancies.

^b^ Columns for receipt of imaging examination are not mutually exclusive of other imaging modalities.

^c^ If gestational age was missing, 40 weeks’ gestation was assumed.

^d^ Race and ethnicity information is not routinely collected in Ontario, Canada.

We found considerable variation across sites in the percentage of pregnancies with imaging, specifically 0.4% to 1.1% for CT, 0.4% to 1.4% for MRI, 3.1% to 8.0% for conventional radiography, 0.2% to 0.5% for angiography and fluoroscopy, and 0.1% to 0.3% for nuclear medicine. Ontario had a lower percentage of pregnancies with CT and MRI than any US site (Table 1). Overall, 5.3% of pregnant women in US sites and 3.6% in Ontario underwent imaging with ionizing radiation, and 0.8% in US sites and 0.4% in Ontario underwent CT.

Imaging with CT was most common in women younger than 20 years or aged 40 years or older, whereas MRI was the most frequent in women aged 40 years or older (Table 1). All imaging modalities were more commonly used in women who gave birth preterm than in those with full-term births. Imaging rates with CT and MRI increased over time; CT increased from 0.25% during 1996 to 2000 to 0.59% of pregnancies during 2011 to 2016, and MRI increased from 0.11% to 0.74% in the same periods. Over the 21-year study period, CT rates increased 3.7-fold in the US sites and 2.0-fold in Ontario. In contrast, radiography, angiography and fluoroscopy, and nuclear medicine imaging rates remained relatively stable over the study period.

In the United States, imaging rates with CT, MRI, and radiography were higher among black women (1.49% of pregnancies for CT, 0.65% for MRI, and 6.69% for radiography) and Native American women (1.16% for CT, 0.73% for MRI, and 5.90% for radiography), compared with white women (0.74% for CT, 0.59% for MRI, and 4.44% for radiography). In contrast, Asian women had lower rates of CT (0.46%) and MRI (0.31%). No significant racial or ethnic differences were observed for angiography and fluoroscopy or nuclear medicine.

### Imaging Rates Over Time

**Figure 1.**
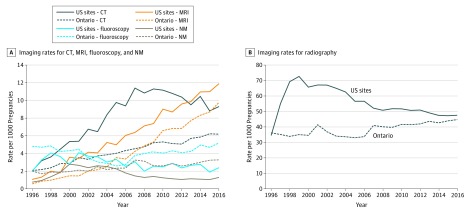
Use Rates of Imaging Modalities in Pregnant Women by Birth Year of Child, US Sites and Ontario, Canada CT indicates computed tomography; MRI, magnetic resonance imaging; and NM, nuclear medicine.

Figure 1 shows imaging rates by each modality over time. In the US sites, CT use rates increased from 2.0 examinations/1000 pregnancies in 1996 to 11.4/1000 pregnancies in 2007, remained stable through 2010, and then decreased to 9.3/1000 pregnancies in 2016. In Ontario, overall CT rates were approximately half those of the US sites, but increased from 2.0/1000 pregnancies in 1996 to 6.2/1000 pregnancies in 2016, which was 33% lower than the US rates. Rates of MRI use also increased steadily over time, from 1.0/1000 pregnancies to 11.9/1000 pregnancies in the United States and from 0.5/1000 pregnancies to 9.8/1000 pregnancies in Ontario, and surpassed CT in 2013 in the United States and in 2008 in Ontario. Radiography use rates increased in the United States from 34.5/1000 pregnancies in 2006 to 72.6/1000 pregnancies in 1999 and then decreased to 47.6/1000 pregnancies in 2016. There was an increase of more than 60% in the use of radiography (primarily chest radiography) from 1996 to 1999 at 1 US site (eFigure in the Supplement). In contrast, in Ontario, radiography use rates increased from 36.2/1000 pregnancies in 1996 to 44.7/1000 pregnancies in 2016, and by 2016, rates were very similar between the United States and Ontario. In the United States, use rates for angiography and fluoroscopy increased from 2.0/1000 pregnancies in 1996 to 4.1/1000 pregnancies in 2001 and then decreased to 2.4/1000 pregnancies in 2016. In Ontario, angiography and fluoroscopy use rates decreased from 4.8/1000 pregnancies in 1996 to 2.6/1000 pregnancies in 2005 and then increased to 5.2/1000 pregnancies in 2016, more than double the US 2016 rate. Nuclear medicine use decreased in the United States from 2003 onward, but increased in Ontario, with rates 2.5 times higher in Ontario (3.3/1000 pregnancies) than in the United States (1.3/1000 pregnancies) by 2016.

### Relative Rates of Imaging Use

After accounting for study site, maternal age, gestational age, and year of birth, adjusted RRs of imaging (Table 2) were consistent with the distributions seen in percentages of pregnancies with imaging (Table 1). Compared with Ontario, imaging rates were significantly different across the US sites, even after accounting for maternal age and gestational age. There were approximately 2- to 3-fold differences in RRs of each imaging modality between the highest and lowest sites across all modalities (1.42-2.78 for CT, 0.73-2.76 for MRI, 1.32-2.53 for radiography, 0.46-1.46 for angiography and fluoroscopy, and 0.47-1.46 for nuclear medicine). Sites in the United States with high rates of imaging with one modality (eg, sites 1 and 5 for CT) also tended to have high rates of imaging with other modalities (MRI, radiography, and nuclear medicine).

**Table 2.** Adjusted Relative Rates and 95% Confidence Intervals of All Imaging for Each Imaging Modality, US Sites and Ontario, Canada

Variable	Adjusted Relative Rate (95% CI)^a^				
	Computed Tomography	Magnetic Resonance Imaging	Radiography	Fluoroscopy and Angiography	Nuclear Medicine
Study site					
US site					
1	2.78 (2.35-3.28)	1.24 (0.96-1.60)	2.53 (2.23-2.87)	1.46 (1.13-1.88)	1.46 (1.07-1.99)
2	1.42 (1.18-1.70)	0.91 (0.72-1.14)	2.30 (2.07-2.55)	0.46 (0.33-0.66)	0.69 (0.48-0.98)
3	1.89 (1.69-2.12)	1.13 (0.98-1.29)	1.32 (1.20-1.45)	0.73 (0.60-0.89)	0.79 (0.63-1.00)
4	2.15 (1.93-2.39)	1.49 (1.31-1.69)	1.52 (1.39-1.67)	1.10 (0.94-1.29)	0.73 (0.57-0.93)
5	2.48 (2.26-2.71)	2.76 (2.54-3.00)	1.34 (1.23-1.47)	1.38 (1.20-1.59)	1.14 (1.02-1.29)
6	1.76 (1.68-1.85)	0.73 (0.69-0.79)	1.32 (1.27-1.37)	0.51 (0.47-0.56)	0.47 (0.42-0.53)
Ontario, Canada	1 [Reference]	1 [Reference]	1 [Reference]	1 [Reference]	1 [Reference]
Maternal age at start of pregnancy, y					
≤19	1.42 (1.31-1.53)	0.99 (0.89-1.11)	1.52 (1.43-1.61)	1.06 (0.94-1.19)	1.22 (1.07-1.40)
20-29	1 [Reference]	1 [Reference]	1 [Reference]	1 [Reference]	1 [Reference]
30-39	0.77 (0.74-0.81)	1.03 (0.99-1.08)	0.89 (0.86-0.92)	0.99 (0.94-1.05)	0.85 (0.79-0.91)
≥40	1.04 (0.93-1.17)	1.38 (1.23-1.54)	1.45 (1.33-1.57)	1.55 (1.35-1.78)	1.17 (0.97-1.41)
Gestational age of neonate at birth, wk^b^					
24-36 (Preterm)	2.15 (2.02-2.30)	3.03 (2.85-3.22)	1.66 (1.57-1.76)	3.12 (2.89-3.37)	1.58 (1.40-1.78)
37-40 (Full-term)	1 [Reference]	1 [Reference]	1 [Reference]	1 [Reference]	1 [Reference]
41-43 (Late-term)	0.77 (0.71-0.83)	0.75 (0.69-0.82)	0.93 (0.88-0.99)	0.72 (0.64-0.81)	0.77 (0.68-0.87)
Year of neonate’s birth					
1996-2000	1 [Reference]	1 [Reference]	1 [Reference]	1 [Reference]	1 [Reference]
2001-2005	1.62 (1.50-1.75)	2.17 (1.93-2.45)	1.04 (0.99-1.09)	0.72 (0.67-0.78)	1.26 (1.13-1.39)
2006-2010	2.21 (2.06-2.38)	4.36 (3.90-4.87)	1.03 (0.98-1.07)	0.72 (0.67-0.78)	1.33 (1.20-1.47)
2011-2016	2.40 (2.23-2.57)	7.52 (6.76-8.37)	1.08 (1.03-1.13)	0.85 (0.79-0.91)	1.36 (1.23-1.50)
Race of mother at US sites^c^					
White	1 [Reference]	1 [Reference]	1 [Reference]	1 [Reference]	1 [Reference]
Asian	0.67 (0.63-0.72)	0.59 (0.54-0.64)	1.02 (0.98-1.05)	0.69 (0.63-0.77)	0.86 (0.77-0.97)
Black	2.10 (1.97-2.24)	1.19 (1.09-1.30)	1.64 (1.57-1.71)	1.19 (1.05-1.35)	1.95 (1.72-2.21)
Hawaiian/Pacific Islander	1.12 (0.95-1.32)	0.71 (0.58-0.88)	0.99 (0.90-1.08)	1.12 (0.86-1.46)	0.99 (0.74-1.34)
Native American	1.42 (1.16-1.75)	1.12 (0.87-1.44)	1.33 (1.17-1.51)	1.41 (1.03-1.93)	1.24 (0.82-1.87)
Unknown or not reported	0.86 (0.80-0.92)	0.74 (0.68-0.80)	0.88 (0.84-0.92)	0.72 (0.65-0.81)	0.99 (0.87-1.13)
Ethnicity of mother at US sites^c^					
Not Hispanic	1 [Reference]	1 [Reference]	1 [Reference]	1 [Reference]	1 [Reference]
Hispanic	1.21 (1.13-1.29)	1.13 (1.04-1.22)	1.17 (1.12-1.21)	1.10 (1.00-1.22)	0.96 (0.85-1.09)
Unknown or not reported	0.98 (0.90-1.06)	0.94 (0.86-1.02)	1.06 (1.01-1.12)	0.93 (0.82-1.04)	0.96 (0.83-1.11)

^a^ All estimates were mutually adjusted for other variables in the Table except race and ethnicity. Estimates for race and ethnicity were adjusted for all other variables in the Table.

^b^ If gestational age was missing, 40 weeks’ gestation was assumed.

^c^ Race and ethnicity information is not routinely collected in Ontario, Canada.

Compared with women aged 20 to 29 years, CT imaging was more common among women younger than 20 years (RR, 1.42; 95% CI, 1.31-1.53), whereas MRI was more frequent in women aged 40 years or older (RR, 1.38; 95% CI, 1.23-1.54) (Table 2). Compared with women with full-term birth (37-40 weeks), all imaging modalities were used more often for women with preterm birth (24-36 weeks), with RRs of 1.66 to 3.12.

Persistent racial and ethnic differences in imaging rates were observed even after adjustment for potential confounders. Compared with white women, use rates for all imaging modalities were significantly higher among black women (RRs, 1.19-2.10), and Native American women had significantly higher rates of CT (RR, 1.42; 95% CI, 1.16-1.75) and radiography (RR, 1.33; 95% CI, 1.17-1.51). Compared with white women, Asian women had lower rates of CT (RR, 0.67; 95% CI, 0.63-0.72), MRI (RR, 0.59; 95% CI, 0.54-0.64), and fluoroscopy (RR, 0.69; 95% CI, 0.63-0.77). Compared with non-Hispanic women, Hispanic women had significantly higher rates of CT (RR, 1.21; 95% CI, 1.13-1.29), MRI (RR, 1.13; 95% CI, 1.04-1.22), and radiography (RR, 1.17; 95% CI, 1.12-1.21).

### Imaging Rates by Anatomic Area

For imaging rates by anatomic area (Table 3; eTable in the Supplement), the increase in CT imaging was most apparent for chest CT. At the US sites, the rate increased from 0.2/1000 pregnancies in 1996 to 2000 to 4.0/1000 pregnancies in 2011 to 2016, and rates in Ontario increased from 0.1/1000 pregnancies to 2.1/1000 pregnancies in the same periods.

**Table 3.** Computed Tomography, Magnetic Resonance Imaging, Radiography, and Fluoroscopy and Angiography Use Rates per 1000 Pregnancies Across 5-Year Intervals of Child’s Birth by Anatomic Region and Geographic Site

Modality, Site	Use Rate/1000 Pregnancies																			
	All Anatomic Regions				Abdomen and Pelvis				Chest				Head and Brain				Other Regions (Extremity, Neck, Spine, Unknown)^a^			
	1996-2000	2001-2005	2006-2010	2011-2016	1996-2000	2001-2005	2006-2010	2011-2016	1996-2000	2001-2005	2006-2010	2011-2016	1996-2000	2001-2005	2006-2010	2011-2016	1996-2000	2001-2005	2006-2010	2011-2016
Computed tomography																				
US sites	3.6	8.1	12.1	10.6	0.9	2.0	2.4	2.1	0.2	1.4	4.4	4.0	2.1	4.3	4.5	3.5	0.4	0.5	0.8	1.0
Ontario, Canada	2.8	4.0	5.3	6.2	0.2	0.5	0.7	0.8	0.1	0.6	1.5	2.1	1.9	2.2	2.1	2.3	0.2	0.4	0.4	0.5
Magnetic resonance imaging																				
US sites	4.0	5.3	8.9	12.8	1.1	1.3	2.9	5.5	0.4	0.1	0.2	0.2	1.4	2.3	3.3	4.3	1.1	1.6	2.4	2.8
Ontario, Canada	1.5	3.5	6.6	9.8	0.1	0.8	2.2	3.6	NA	0.1	0.1	0.2	0.5	1.0	1.6	2.7	0.3	0.5	1.0	1.5
Radiography																				
US sites	76.0	76.6	62.6	58.6	3.3	3.9	3.4	3.5	38.8	38.1	30.0	29.3	4.9	3.2	2.8	1.0	28.9	31.4	26.5	24.8
Ontario, Canada	36.2	41.4	41.7	41.7	3.7	2.9	4.6	5.2	10.5	12.7	16.8	20.0	2.1	1.4	1.0	0.8	18.6	18.7	16.9	17.1
Angiography and fluoroscopy																				
US sites	3.4	4.6	3.3	3.5	2.3	3.0	1.7	1.7	0.4	0.4	0.2	0.3	0.03	0.1	0.1	0.01	0.7	1.2	1.3	1.4
Ontario, Canada	4.9	4.4	4.2	5.2	4.0	2.4	2.4	2.9	0.2	0.3	0.5	0.6	0.1	0.3	0.1	0.1	0.2	0.2	0.7	1.0
Nuclear medicine																				
US sites	3.1	3.5	1.7	1.3					1.5^b^	2.3^b^	1.1^b^	0.9^b^					1.6^c^	1.2^c^	0.6^c^	0.4^c^
Ontario, Canada	2.0	2.5	3.2	3.3					0.8^b^	1.5^b^	2.3^b^	2.5^b^					1.0^c^	0.7^c^	0.5^c^	0.4^c^

Abbreviation: NA, not applicable (no observations).

^a^ See eTable in the Supplement for use rates for each individual other anatomic region.

^b^ Ventilation perfusion imaging (chest).

^c^ Other nuclear medicine, including heart nuclear medicine.

**Figure 2.**
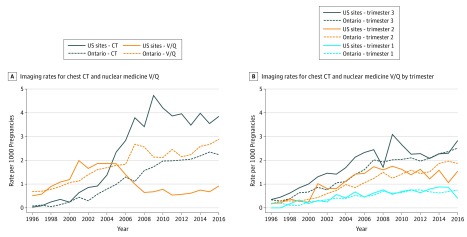
Use Rates of Chest Computed Tomography (CT) and Nuclear Medicine Ventilation-Perfusion (V/Q) Imaging by Trimester, US Sites and Ontario, Canada

In the United States, the use of chest CT and V/Q scanning increased steadily until around 2004 when these modalities were used approximately equally (1.9/1000 pregnancies; Figure 2A). Afterward, their use started to diverge, with rapid growth in the use of chest CT and a rapid decrease in V/Q scanning. By 2016, the United States was using nearly 4 times as many chest CT scans (3.9/1000 pregnancies) as V/Q scans (0.9/1000 pregnancies). In Ontario, rates for both chest CT and V/Q scans increased steadily from 1996 (<1/1000 pregnancies) to 2016 (2.2-2.9/1000 pregnancies), with rates of V/Q scans always higher than those for chest CT (Figure 2A). The use of both CT and V/Q scanning was greater in the second and third trimesters of pregnancy in both the US sites and Ontario, and the largest growth in imaging occurred in the third trimester (Figure 2B).

## Discussion

Among pregnant women, the use of medical imaging with ionizing radiation greatly increased from 1996 to 2016 in our large study of more than 3.4 million pregnancies in 6 US health systems and the provincial health system of Ontario, Canada. Overall, 5.3% of pregnant women in the US sites and 3.6% in Ontario underwent imaging with ionizing radiation, primarily radiography (4.6% in the United States and 3.1% in Ontario) and CT (0.8% in the United States and 0.4% in Ontario). Over the 21-year study period, CT rates increased 3.7-fold in the US sites and 2.0-fold in Ontario, whereas the use of other imaging modalities with ionizing radiation decreased. In Ontario, imaging with ionizing radiation was used less frequently than in the US sites, whereas MRI, which does not use radiation, was used more frequently. We found only 1 study^6^ of maternal imaging trends in the literature. This study from a single US institution reported that rates increased by 25% between 1997 and 2006. Our multisite study showed a 200% increase across the same period.

There are multiple factors that might explain the increasing rates of imaging observed over our 21-year study period. They include advances in imaging technology, patient- and physician-generated demand, defensive medical practices,^24^ medical uncertainty, and unanticipated modifying factors. In fact, we observed much higher rates of radiography at 1 US site in the late 1990s associated with a policy of evaluating positive tuberculosis skin test results with chest radiography.^25^ When comparing our reported imaging rates for pregnant women over time, the trends are similar to those in the general adult population, although the magnitude of rates is lower in pregnant women. For example, in 2010 for CT, rates in pregnant women were 11.2 examinations/1000 pregnancies compared with 149 examinations/1000 nonpregnant individuals. However, in both groups, rates increased 3 to 5 times between 1996 and 2010.^21^

Pregnant women are at increased risk of pulmonary embolism, which is one of the most common causes of maternal death in developed countries and is estimated to occur in 1/1000 pregnancies.^26,27^ Both V/Q scan and chest CT are used to diagnose pulmonary embolism and both have relatively low radiation exposure to the fetus (0.32-0.64 mGy and 0.0033-0.1308 mGy, respectively).^28^ However, maternal radiation exposure associated with chest CT, especially to the breast, is higher compared with that associated with V/Q scan.^27^ Diagnostic criteria for the management of suspected pulmonary embolism in pregnant women have largely been gleaned from studies in nonpregnant populations and retrospective studies and, thus, remain inconsistent and under debate.^29^ Most guidelines recommend the use of V/Q scan over chest CT, primarily to minimize maternal radiation exposure.^30^ However, a recent study^29^ in 2018 reported high performance of a CT-based diagnostic strategy. In our study, although we did not have indication information for the imaging examination, we observed higher rates of chest CT than V/Q imaging in the United States and the opposite trend in Ontario.

We observed that black, Native American, and Hispanic women; women younger than 20 years and those aged 40 years and older; and women who gave birth preterm underwent significantly more imaging with ionizing radiation, most notably CT, whereas Asian women underwent significantly less imaging. It is unclear why there are possible racial and age disparities in the receipt of medical imaging by pregnant women. We hypothesize that minority women and younger women, compared with their counterparts, might be seen more often in emergency settings for abdominal pain where imaging is performed for clinical workup. However, the reasons for more frequent imaging in women who gave birth preterm are more complex because pregnancy complications could have led to both increased imaging and preterm delivery. Further investigation of variation in imaging use across patient subpopulations is needed to ensure that radiation is used only when necessary in pregnant women. The in utero dose received by the fetus must also be considered given the higher radiosensitivity of fetal tissues, especially during the first trimester. Radiation susceptibility is thought to be highest at this gestational stage as a result of organogenesis.^31^

To our knowledge, our study is the largest and most contemporary to date to characterize medical radiation use over a 21-year span in pregnant women who gave birth to a live neonate after at least 24 weeks of gestation. It is, to our knowledge, the first international comparison of imaging use for pregnant women between the United States and Canada. It is also a comprehensive assessment of all imaging modalities, except ultrasonography, with data obtained from health plan electronic data sources. Furthermore, our study provides a unique representation of the US population insured by similar integrated health care and commercially insured systems, and includes all residents of Ontario, Canada, eligible for provincial health coverage.

### Limitations

We investigated medical imaging in pregnant women who gave birth to a live neonate after 24 weeks’ gestational age. Therefore, no inferences can be made about imaging done for pregnancies that did not result in a live birth. In part, we did not investigate these pregnancies because of incomplete capture of this information across sites. As for some pregnant women who might have received care outside the health plan or province, we minimized this potential exposure misclassification by including only women in our cohort who were enrolled throughout their entire pregnancy. Outside claims for imaging services are routinely included in each US health plan’s electronic health record and are thus trackable as part of the patient’s clinical care. Given that health care in Ontario is organized as a single-payer system, examinations outside the system would be exceedingly rare. Our estimates may be conservative because we did not count multiple examinations for the same modality and anatomic area that occurred on the same day. To identify true repeat examinations on the same day would require resource-intensive medical record review. In addition, we did not have access to complete data on indications for the imaging examinations.

## Conclusions

Rates of medical imaging of pregnant women have greatly increased since 1996 and are higher in the United States than in Canada. The use of CT started declining in the United States in 2007 but continued to increase in Ontario. The use of MRI is growing and has surpassed CT use in both countries, likely because it improves assessment of fetal anomalies and does not expose the mother or fetus to ionizing radiation. Assuming that the experience of these health plans is typical of US and Canadian practice, we observed a shift in imaging practice patterns over the past 10 years in the direction of tempering exposure to imaging procedures involving ionizing radiation in support of other modalities. Continuing to monitor imaging rates in pregnant women is important to avoid unnecessary testing and ionizing radiation exposure to the woman and fetus.

## Notes

Supplement. **eTable. **Use Rates of CT, MRI, Radiography, and Fluoroscopy and Angiography per 1000 Pregnancies by 5-Year Intervals of Child’s Birth, Among Other Anatomic Category, US Sites and Ontario, Canada **eFigure.** Use Rates of Radiography in Pregnant Women by Birth Year of Child and Individual Study Site
