# Supplementary material for: Trends in Medical Imaging During Pregnancy in the United States and Ontario, Canada, 1996 to 2016
Source: JAMA Netw Open. 2019 Jul 24;2(7):e197249. doi: 10.1001/jamanetworkopen.2019.7249 (PMC6659354; doi:10.1001/jamanetworkopen.2019.7249)
Supplement: Supplement. — eTable. Use Rates of CT, MRI, Radiography, and Fluoroscopy and Angiography per 1000 Pregnancies by 5-Year Intervals of Child’s Birth, Among Other Anatomic Category, US Sites and Ontario, Canada eFigure. Use Rates of Radiography in Pregnant Women by Birth Year of Child and Individual Study Site [file jamanetwopen-2-e197249-s001.pdf]

Supplementary Online Content

Kwan ML, Miglioretti DL, Marlow EC, et al. Trends in medical imaging during pregnancy in the United States and Ontario, Canada, 1996-2016. *JAMA Netw Open*. 2019;2(7):e197249. doi:10.1001/jamanetworkopen.2019.7249

eTable. Use Rates of CT, MRI, Radiography, and Fluoroscopy and Angiography per 1000 Pregnancies by 5-Year Intervals of Child's Birth, Among Other Anatomic Category, US Sites and Ontario, Canada

eFigure. Use Rates of Radiography in Pregnant Women by Birth Year of Child and Individual Study Site

This supplementary material has been provided by the authors to give readers additional information about their work.

© 2019 Kwan ML et al. *JAMA Network Open*

eTable. Use Rates of CT, MRI, Radiography, and Fluoroscopy and Angiography per 1000 Pregnancies by 5-Year Intervals of Child's Birth, Among Other Anatomic Category, U.S. Sites and Ontario, Canada

**Modality, Site**	**Extremity**				**Neck**				**Spine**				**Unknown**			
	**Year of Birth**				**Year of Birth**				**Year of Birth**				**Year of Birth**			
	**1996-2000**	**2001-2005**	**2006-2010**	**2011-2016**	**1996-2000**	**2001-2005**	**2006-2010**	**2011-2016**	**1996-2000**	**2001-2005**	**2006-2010**	**2011-2016**	**1996-2000**	**2001-2005**	**2006-2010**	**2011-2016**
**Computed Tomography**																
U.S. Sites	0.04	0.10	0.10	0.06	0.07	0.10	0.19	0.23	0.16	0.22	0.43	0.67	0.15	0.08	0.07	0.01
Ontario, Canada	0.06	0.09	0.13	0.09	0.00	0.05	0.08	0.14	0.18	0.22	0.23	0.23	0.05	0.00	0.00	0.00
**Magnetic Resonance Imaging**																
U.S. Sites	0.63	0.55	0.90	1.16	n/a[1]	n/a	n/a	n/a	0.46	1.04	1.40	1.43	0.00	0.06	0.15	0.17
Ontario, Canada	0.08	0.16	0.28	0.42	n/a	n/a	n/a	n/a	0.16	0.30	0.71	1.13	0.09	0.04	0.00	0.00
**Radiography**																
U.S. Sites	23.17	23.83	21.18	21.34	23.17	23.83	21.18	21.34	0.16	0.18	0.25	0.23	4.00	4.96	4.15	3.03
Ontario, Canada	14.83	14.95	14.07	14.48	14.83	14.95	14.07	14.48	0.20	0.52	0.15	0.20	3.56	3.25	2.67	2.38
**Angiography and Fluoroscopy**																
U.S. Sites	0.10	0.03	0.02	0.02	0.10	0.03	0.02	0.02	0.02	0.10	0.01	0.04	0.03	0.31	0.23	0.20
Ontario, Canada	0.03	n/a	0.06	0.07	0.03	n/a	0.06	0.07	0.08	0.05	0.04	0.00	0.14	0.15	0.49	0.62

[a] n/a = no observations

© 2019 Kwan ML et al. *JAMA Network Open*

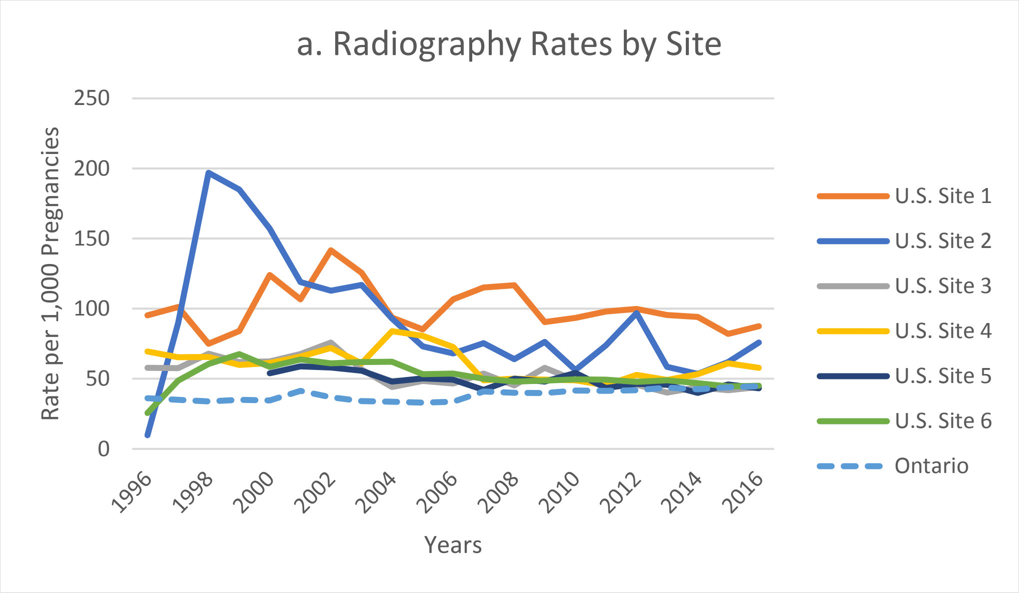

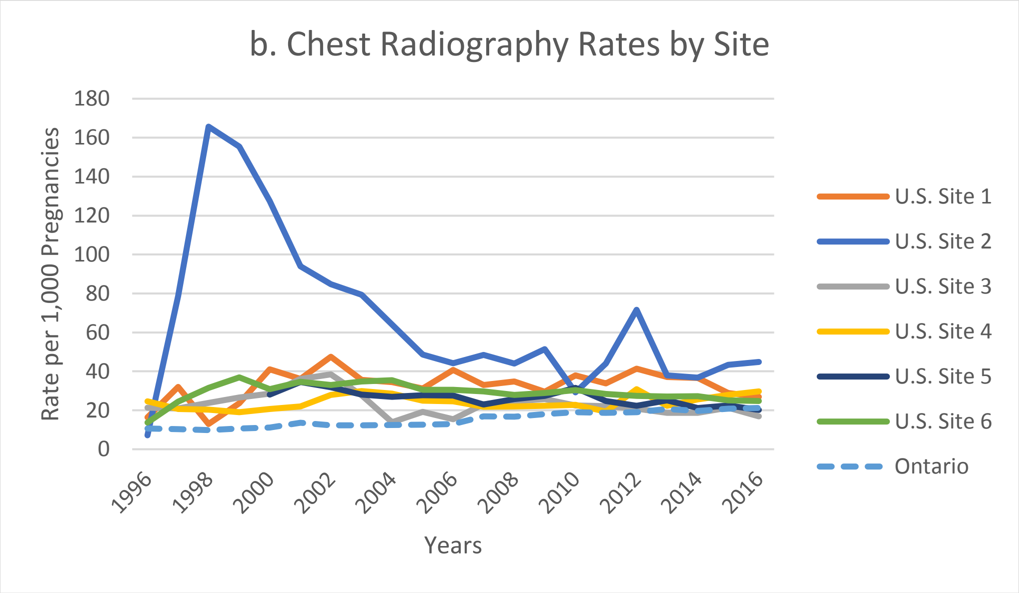

eFigure . Use Rates of Radiography in Pregnant Women by Birth Year of Child and Individual Study Site

© 2019 Kwan ML et al. *JAMA Network Open*
